# Arabic Patient-Reported Measures of Activity and Participation for Children: A Systematic Review of Psychometric Properties

**DOI:** 10.3390/children10091566

**Published:** 2023-09-18

**Authors:** Mohammed S. Alghamdi, Enas Alharbi, Rawan Alghamdi, Ahmed S. Alhowimel, Aqeel M. Alenazi, Mohammed M. Alshehri, Bader A. Alqahtani, Abdulaziz Awali

**Affiliations:** 1 Department of Physical Therapy, College of Applied Medical Sciences, Umm Al-Qura University, Makkah 21955, Saudi Arabia; 2 Department of Health and Rehabilitation Sciences, Prince Sattam Bin Abdulaziz University, Al-Kharj 11942, Saudi Arabia; 3 Department of Physical Therapy, College of Applied Medical Sciences, Jazan University, Jazan 45142, Saudi Arabia

**Keywords:** Arabic patient-reported measure, activity, participation, psychometrics, children

## Abstract

Aim: To systematically review measurement properties of Arabic patient-reported outcome measures (PROMs) that assess activity and participation in children with and without health conditions. Method: Four databases were searched. Arabic PROMs with focus on activity and/or participation constructs were selected. Data on measurement properties were extracted and the methodological quality of the studies was assessed by COnsensus-based Standards for the selection of health Measurement Instruments (COSMIN) risk of bias checklist. Result: Of the total 149 articles screened, only 10 studies involving 10 measures that assessed activity and/or participation in children with or without health conditions were included. The focus of all PROMs is primarily on the activity of daily living at home and/or school, but dimensions of measurement differed across PROMs. None of the PROMs demonstrated sufficient properties for all psychometrics. The most studied psychometric property was internal consistency, whereas the least studied psychometric property was structural validity. Responsiveness was not investigated in any of the studies included. Conclusions: Despite the presence of Arabic PROMs on activity and participation for children, none of the reviewed measures satisfied all psychometric properties. Clinicians and researchers are encouraged to carefully select PROMs that are psychometrically sound and appropriate for the construct being measured.

## 1. Introduction

Since the emergence of the International Classification of Functioning and Disability (ICF) in early 2000 [1], there has been an increasing interest in research and clinical practice for understanding activity and participation for children with and without health conditions. The ICF defines “activity” as the execution of a task by an individual, while “participation” concerns a person’s involvement in life situations [1]. However, lack of clarity is still existing in the literature between these terms due to overlaps in description, varied interpretations among professionals and cultures, and challenges in operationalizing these concepts for measurement [2,3,4]. Measurement tools related to activity and participation generally involve selection of items from nine domains described in the ICF manual [1,3]. These domains are learning and applying knowledge, general tasks and demands, communication, mobility, self-care, domestic life, interpersonal interactions and relationships, major life areas, and community, social and civic life [1].

Activity and participation are important constructs for human functioning in daily life and important rehabilitation outcomes for children with health conditions [3,5,6]. Depending on the purpose of the tool, the dimensions of measurement may be related to frequency and diversity, enjoyment, difficulty, independence, or satisfaction [2,3]. The availability of psychometrically sound measures for activity and participation for children would enable stakeholders (e.g., researchers, clinicians, and families) to assess, monitor and tailor services to improve these outcomes [5]. Patient-reported outcome measures (PROMs) are important tools that allow patients to directly report on their health-related status through a standardized set of items or questions. In general, PROMs are useful for understanding many aspects, including but not limited to patient experience of illness, functional performance, and quality of life [7]. The utility of PROMs goes beyond direct patient care and contributes to improving the cost-effectiveness of services and providing data to review policies and practices related to patient care [8]. For children, there is a wide range of PROMs available to assess children’s health-related constructs, including activity and participation, especially for children with health conditions [9]. Although PROMs are typically used to assess patient health status directly, the method of administration for the pediatric population is mostly carried out by proxy, i.e., a parent or caregiver reporting on the behavior of the child. Although agreement between children’s reports and parents’ proxy reports can be influenced by a wide range of factors such as the child’s age and type of illness [10,11], parents’ proxy reports in pediatric practice remain instrumental in the clinical decision-making process.

The existence of robust psychometric properties in a specific PROM is a critical factor determining its use in both research and clinical practice [12]. The COnsensus-based Standards for the selection of health Measurement Instruments (COSMIN) is an initiative that aims to advance the science of measurement development through the creation of methodological guidelines and tools to assist in the development of PROMs [13]. The COSMIN initiative has reached consensus on the taxonomy of measurement properties, the relationship between these properties, and their definitions [14]. The domains of measurement properties include reliability, “the degree to which the measurement is free from measurement error”; validity, “the degree to which a PROM measures the construct(s) it is supposed to measure”; and responsiveness, “the ability of a PROM to detect change over time in the construct to be measured” [14].

Worldwide, there are 23 countries in which Arabic is the primary language, with approximately 420 million Arabic speakers [15]. Al-Muqiren et al. [16] reported that the major barrier for utilizing patient-reported measures in Arab countries is that existing measures are only available in English. Cross-cultural adaptation is a process in which existing valid and reliable measures developed in one language are translated and adapted to another language [17]. Despite the increasing amount of research on cross-cultural adaptation of health-related measures, there is still a limited number of measures in the area of activity and participation for children. Albawardi and colleagues [18] described the development of an open-access database of Arabic Health Measures, which is a Saudi-based initiative developed to provide an access portal for Arabic health-related measures and enhance uptake of these measures. The authors reported that the majority of studies concerning measures for adults and children in the database discussed psychometric testing, but further investigation of the quality of the measurement properties is needed [18]. The aim of this study, therefore, was to critically appraise, compare and summarize the quality of the measurement properties of existing Arabic patient-reported outcome measures that assess activity and participation for children.

## 2. Methods

The design of this study is a systematic review. We used the Preferred Reporting Items for Systematic Review and Meta-Analyses (PRISMA) in reporting this systematic review [19].

### 2.1. Search Strategy

A comprehensive literature search was used to retrieve studies concerning development or adaptation of Arabic PROMs for children that focus on activity and participation. Four major databases (MEDLINE via PubMed, Web of Science, PsycInfo, and Arab Health Measures) were searched. The search strategy used a combination of key terms that followed PICO format: (1) pediatric population (up to 18 years of age), (2) Arabic measures or tools related to activity and participation terms, and (3) terms relevant to measures and psychometric properties. Given that activity and participation have been broadly defined in the ICF framework, the search was inclusive of all nine domains of the “activity and participation” described in the ICF manual [1]. Databases were searched without any time limits on publications. The search was conducted during February 2023. The protocol for this review was prospectively registered in PROSPERO (registration number CRD42023395458).

### 2.2. Inclusion/Exclusion Criteria

The eligibility criteria were (1) PROMs related to activity and participation for children with and without health conditions, (2) PROMs designed for children up to the age of 18 years, (3) original Arabic PROMs or PROMs that have been translated into the Arabic language, and (4) studies should be focused on the evaluation of one or more measurement properties. The exclusion criteria were (1) non-Arabic PROMs, (2) PROMs that were designed as part of other studies (e.g., RCTs), and (3) PROMs in which activity and participation were not clearly defined.

### 2.3. Instrument and Study Selection

Based on the eligibility criteria, two of the authors (RA and EA) independently screened study titles and abstracts to determine relevance. Once relevance had been determined, the full texts were read by the two authors (RA and EA) to determine which PROMs should be used to assess activity and participation in children, and to extract relevant data.

### 2.4. Extraction and Synthesis of Measurement Properties

Measurement properties of PROMs were extracted from the included studies per the COSMIN guidelines. Two of the authors (RA and EA) independently extracted data which included characteristics of participants, psychometric properties (content validity, cross-cultural validity, reliability and responsiveness). Another author (M.S.A) was invited to resolve any disagreement between the two authors during the data extraction. Authors of studies included in the review were contacted if missing information was found.

### 2.5. Rating Methodological Quality of Studies

To assess the methodological quality of studies examining measurement properties, we used the COSMIN risk of bias checklist [13]. The methodological quality of the study was rated on a four-point rating scale: very good, adequate, doubtful, and inadequate. The lowest rating of any psychometric properties was taken. The reliability of the COSMIN risk of bias checklist was supported [13]. Psychometric properties were rated using the updated criteria for good measurement properties [20]. Each measurement property was rated as sufficient (denoted with a “+” sign), insufficient (denoted with a “−” sign), or indeterminate (denoted with a “?” mark).

## 3. Results

**Figure 1:**
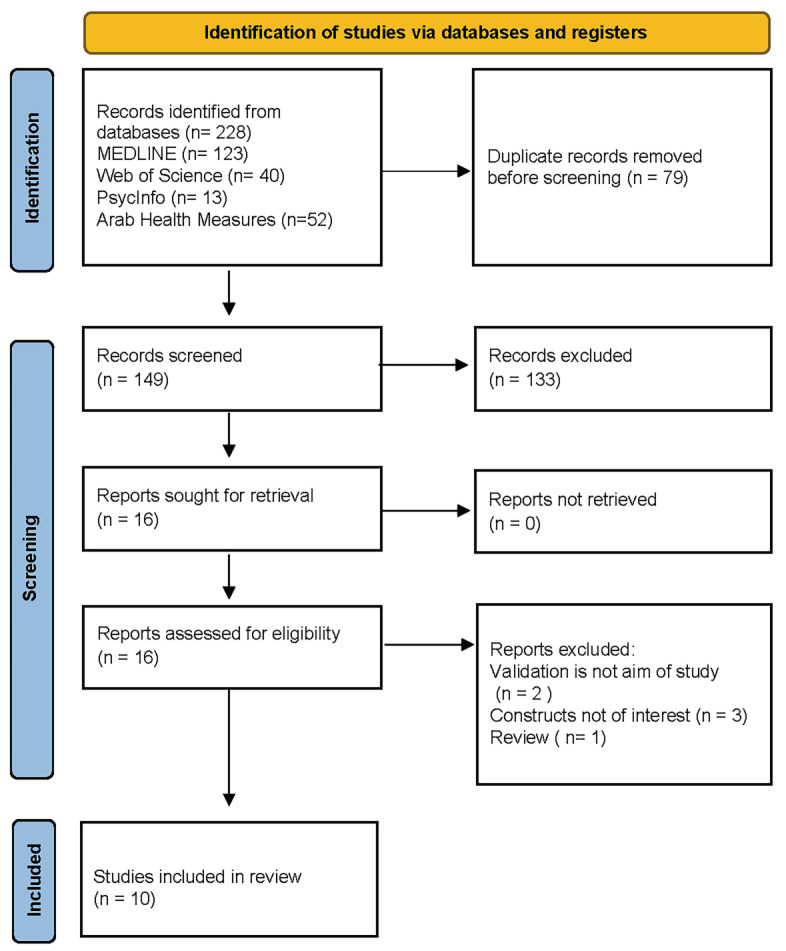
Flowchart of literature search.

The search yielded 228 unique records and 149 remained after removing duplicates. We retrieved 16 full-text articles after screening titles and abstracts. A total of 10 articles met the eligibility criteria and were included in the systematic review. Figure 1 depicts the flowchart of the literature search.

Each of the studies (*n* = 10) represents a unique PROM that is focused on activity and/or participation for children with or without health conditions. The PROMs included in the review were validated for different populations: typically developing children, children with obesity/overweight, children with cerebral palsy (CP), and children with juvenile idiopathic arthritis. The 10 measures included in the review were studied in six different countries: Saudi Arabia (*n* = 4), Jordan (*n* = 2), United Arab Emirates (*n* = 1), Oman (*n* = 1), Morocco (*n* = 1), and Tunisia (*n* = 1). Half of the measures included were completed by parents’ proxy reports. The focus of all measures was primarily on the activity of daily living at home and/or school. The dimensions of measurement were participation [21,22], functional abilities [23,24,25,26], duration and frequency of physical activity [27,28,29], and self-perception of physical activity [30]. Table 1 provides a description of the PROMs included in the review.

Among all PROMs, the most studied psychometric property was the internal consistency whereas the least studied psychometric property was the structural validity. Responsiveness was not investigated in any of the studies included. Content validity was not described in all of the studies, because the PROMs included were cross-culturally adapted based on original versions. A succinct review of each PROM is given narratively. Table 2 summarizes the rating of the methodological quality of the studies investigating the measurement properties of PROMs. Table 3 summarizes the rating of the psychometric properties of the PROMs included in the review.

### 3.1. Children’s Assessment of Participation and Environment (CAPE) and Preference for Activity and Participation (PAC)

Almasri and colleagues [21] examined the CAPE-PAC in 150 children CP and without CP in Jordan. Children’s age range was between 6 to 18. Internal consistency, structural validity and construct validity were examined. The internal consistency and construct validity were rated sufficient. Structural validity was rated as insufficient given that only a principal component analysis was used. No other psychometric properties were examined.

### 3.2. Pediatric Evaluation of Disability Inventory (PEDI)

Al-Khudair and Al-Eisa [23] examined the cross -cultural applicability of the (PEDI) in 52 typically developing children in Saudi Arabia (aged 1 to 7 years). The only psychometric property examined was the internal consistency, and was rated as sufficient. A unique feature of this study is that the authors compared the average self-care performance of children in Saudi Arabia with that of children in the United States. This comparison was used to support the cross-cultural validity, despite the inexplicit statement from the authors.

### 3.3. Self-Care Domain of Child Engagement in Daily Life (CEDL)

The Arabic version of Self-care Domain of Child Engagement in Daily Life (Selfcare-CEDL) has been studied for its measurement properties in 36 children with CP (aged 1.5–11 years) in Saudi Arabia [24]. Internal consistency and test re-test reliability were sufficient given the values of Cronbach’s alpha and ICC, which were 0.91 and 0.99, respectively. Although the smallest detectable change (SDC) was examined, the measurement error was rated as indeterminate, as the minimal important change (MIC) was not reported.

### 3.4. International Physical Activity Questionnaire for Adolescents (IPAQ-A)

Regaieg and colleagues [27] examined the reliability and validity of an Arabic version of (IPAQ-A) in 51 overweight adolescents in Tunisia with a mean age of 16.8 years. Test re-test reliability was sufficient, given the values of ICC, which were 0.73 to 0.95. A subgroup analysis was carried out between ages and genders, but did not support construct validity. No other psychometric properties were examined.

### 3.5. Arab Teens Lifestyle Study (ATLS) Physical Activity Questionnaire

Al-Hazzaa and colleagues [28] examined the ATLS physical activity questionnaire in 75 typically developing children in Saudi Arabia with mean age of 16.1 years. The internal consistency, construct validity and structural validity were rated indeterminate. Only convergent validity was assessed, but it was rated as indeterminate, as the comparison measure (pedometer) was not considered as gold standard.

### 3.6. Arabic Preschool Activity Card Sort (PACS)

The Arabic version of PACS has been studied for its measurement properties in 151 typically developing children (aged 3–6 years) in Jordan [22]. Internal consistency and test re-test reliability were sufficient given the values of Cronbach’s alpha and ICC, which were 0.859, and 0.976, respectively. The authors use Spearman’s correlation coefficients between subgroups (according to sample age) to assess the construct validity. No other psychometric properties were examined.

### 3.7. Questionnaire l’Activite Physique en Altitude Chez les Enfants (QAPACE)

The QAPACE was originally developed in France. The name of the measure translates to “Quantification of Physical Activity at Altitude in Children”. The Arabic version was validated for 79 typically developing children aged 6 to 9 years old in the United Arab Emirates [29]. Test re-test reliability was rated as insufficient given the values of ICC, which were 0.4 to 0.5. The authors used Spearman’s rank-correlation coefficients between the questionnaire and the pedometer to assess the construct validity. No other psychometric properties were examined.

### 3.8. Perceived Physical Ability Scale for Children (PPASC)

Abd-Elfattah and colleagues [30] examined the psychometric properties of the (PPASC) in 250 typically developing children in Oman with a mean age of 10 years. Internal consistency and structural validity were sufficient given Cronbach’s alpha and CFI values of 0.89 and 0.986, respectively. No other psychometric properties were examined.

### 3.9. Childhood Health Assessment Questionnaire (CHAQ)

The Arabic version of CHAQ has been studied for its measurement properties in 60 children with juvenile idiopathic arthritis (aged 4–16 years) in Morocco [25]. Internal consistency and test re-test reliability were sufficient, given the Cronbach’s alpha and ICC values, which were 0.90 and 0.82, respectively. No other psychometric properties were examined.

### 3.10. ABILHAND-Kids Scale

Alnahdi and colleagues [26] examined the cross-cultural and measurement proprieties of the (ABILHAND-Kids) in 154 children with CP in Saudi Arabia, with a mean age of 7.4 years. Internal consistency and test re-test reliability were sufficient, given the person separation index and ICC values, which were 0.93 and 0.98, respectively. Although the smallest detectable change (SDC) was examined, the measurement error was rated as indeterminate, as the minimal important change (MIC) was not reported. Structural validity was rated as sufficient given that adequate model fit. Spearman’s correlation coefficients were used to support construct validity, and it was rated as sufficient. The authors compared the item hierarchy in the Arabic ABILHAND-Kids to the item hierarchy in the original ABILHAND-Kids. This comparison was used to support cross-cultural validity.

## 4. Discussion

In the present review, we investigated the quality of the measurement properties for existing Arabic PROMs focused on activity and participation in children with and without health conditions. As expected, the lack of clarity noticed in the literature with regard to operational definition and measurement of activity and participation is reflected in the findings of this study [2,3,4]. In this review, we found that all PROMs are focused on different aspects of activity of daily living at home and/or school. However, the dimensions of measurement for PROMs included in this review varied considerably. Four measures were designed to assess the functional abilities of children (mostly the child’s level of independence) for a wide range of activities (e.g., mobility, self-care) [23,24,25,26]. Three measures were designed to assess physical activity with focus on duration and frequency and intensity [27,28,29]. Two measures were designed to assess participation from multiple aspects (e.g., diversity of participation, enjoyment, environmental accommodation) [21,22]. Only one measure was focused on the self-perception of physical activity (level of strength, speed, and coordinative abilities) [30]. Collectively, our findings illuminate the need to adopt evidence-based approaches [2,31] that clearly delineate the constructs of activity and participation, ensuring both coherent definition and sound measurement tools in future studies.

Among the 10 Arabic PROMs included in the review, none demonstrated sufficient properties for all psychometrics described in COMSIN guidelines. Our findings are consistent with the findings of previous systematic reviews that used COMSIN tools to assess the methodological quality of pediatric PROMs for participation [32], upper limb impairments [33], and pain [34]. All reviews concluded that the methodological quality of the PROMs reviewed did not reach sufficient psychometrics properties. Potential factors contributing to this finding may be related to the diversity of populations, sample sizes, and methods used in development of these PROMs. COSMIN guidelines uses the “worst score counts” rule to determine the overall quality of the study, which also contributed to the finding that none of the PROMs demonstrated sufficient quality in our study. The majority of the studies in this review were published before the development of COSMIN guidelines [35], which might explain the low adherence to the measurement criteria included in COSMIN guidelines. Indeterminate ratings of measurement properties resulted if they had not been examined, and if all the information needed to evaluate the quality criteria as good measurement properties were not presented.

Among all PROMs, the most studied psychometric property is the internal consistency, whereas the least studied psychometric property is the structural validity. Internal consistency can by determined with much less effort than structural validity, which requires complex statistical analysis and a large sample size to conduct Rasch analysis. Confirmatory factor analysis, Rasch analyses or analyses based on item response theory required a large sample, which was not the case in the PROMs we reviewed. Most noticeably, responsiveness was not investigated in any of the studies included. It required enough time and intervention to determine its effectiveness. The ABILHAND-Kids scale [26] had the most sufficient rating of the psychometric properties among all PROMs. The authors used the COSMIN risk of bias checklist as a reference in this study, which explains the sufficient ratings of psychometric properties.

All PROMs but ALTS [28] were originally developed in other languages, and required cross-cultural adaptation. Based on COMSIN guidelines for meeting sufficient cross-cultural adaptation, there was need for comparison between the original and translated measures. PEDI and the ABILHAND-Kids scale were the only measures that reported comparison between the population of original and translated measures. Back translation is considered an important step in cross-cultural adaptation; however, it was carried out in three PROMs (PEDI, Self-care-CEDL, and ALBIHAND-Kids). COSMIN guidelines on cross-cultural adaptation were not extensive compared with existing guidelines by Beaton et al. [17]. In the COSMIN guidelines, cross-cultural adaptation was rated sufficient only if there was comparison between the source and target versions of the PROMs, whereas Beaton et al. [17]’s guidelines describe a multi-stage process used to achieve cultural equivalence.

In this review, five of the ten PROMs included were completed by parents’ proxy reports. Agreement between children’s reports and parents’ proxy reports can be influence by the child’s age, illness type and nature, the outcomes being investigated (e.g., symptoms, quality of life, mobility), and the parents’ own perceptions of the outcomes being investigated [10,11]. While young children below the age of eight years have often been evaluated through parents’ proxy reports due to their limited cognitive and language skills, the utility of these methods becomes less effective as children transition into adolescence [36]. Research suggests that there is strong agreement between children’s reports and parents’ proxy reports for physical aspects of health, compared to emotional or social aspects [10]. These findings collectively offer clinicians and researchers perspectives to effectively engage both child and parent in the clinical decision-making process.

The implications of our findings are three-fold. First, clinicians and researchers are encouraged to carefully select PROMs that are psychometrically sound and appropriate for the construct being measured. Second, the findings of our study can be utilized in the Arabic Health Measures database to present the rating of psychometric properties of PROMs included in this study for consumers to assist them in selecting appropriate PROMs. Third, future studies on measurement development should adopt COSMIN guidelines to produce high-quality PROMs with sufficient psychometric properties.

## 5. Limitations

We have relied on the original definition of activity and participation described in the ICF manual and this may have contributed to the heterogeneity of the PROMs included in the study. In addition, despite the fact that we conducted a comprehensive search in four databases, there might be a chance that we missed measures that were published in Arabic or published in journals not indexed in these databases. We were unable to carry out a meta-analysis because it depends on a series of studies on a single measure to produce a point estimate of an effect and measures of the precision of that estimate. Since all of the PROMs in this review were not studied twice, pooling of the data was not possible.

## 6. Conclusions

This review evaluated the quality of Arabic Patient-Reported Outcome Measures (PROMs) concerning activity and participation in children. There is a noticeable lack of clarity in defining and measuring these constructs in the current literature. Among the 10 reviewed PROMs, none met all the criteria set by COMSIN guidelines, with structural validity being the least studied property. The ABILHAND-Kids measure had the most sufficient rating among all measures reviewed. Half of PROMs relied on parents’ proxy reports, which may differ in reliability based on their child’s age and the nature of the outcomes investigated. Hence, clinicians and researchers are encouraged to choose psychometrically sound PROMs for assessing the activity and participation in children with and without health conditions.

## Figures and Tables

**Table 1:** Description of PROMs included in the review.

Study	Instrument	Country	Population	Age	Respondent	Format of Administration	Focus	Dimension of Measurement
Almasri et al. [21]	Children’s Assessment of Participation and Enjoyment (CAPE) and Preferences for Activities of Children (PAC)	Jordan	Children and youth with cerebral palsy (*n* = 75) and children with typical development (*n* = 75)	6–18 years of age	Child	Interview	Recreational, physical, social, skill-based, and self-improvement activities	Participation diversity, frequency, companionship, (with whom), environment (where), enjoyment, and preferences
Al-khudair et al. [23]	Pediatric Evaluation of Disability Inventory	Saudi Arabia	Children with typical development (*n* = 52)	1–7 years of age	Child	Interview	Self-care, mobility, and social activities	Functional skills, caregiver assistance, and environmental modification
Alghamdi et al. [24]	Self-care Domain of Child Engagement in Daily Life (Selfcare-CEDL)	Saudi Arabia	Children with cerebral palsy (*n* = 36)	1.5–11 years of age	Proxy report (by parents)	Paper-and-pencil	Self-care activities	Functional ability with focus on level of child’s independence
Regaieg et al. [27]	International Physical Activity Questionnaire for Adolescents (IPAQ-A)	Tunisia	Overweight and obese adolescents (*n* = 51)	15–18 years of age	Child	Paper-and-pencil	Physical activities in school, transportation, housework, leisure	Duration (minutes) and frequency (days) in last 7 days
Al-Hazzaa et al. [28]	Arab Teens Lifestyle Study (ATLS) Physical Activity Questionnaire	Saudi Arabia	Children with typical development (*n* = 75)	Average age 16.1 ± 1.1 years	Child	Paper-and-pencil	Physical activities in transport, household, fitness and sports activities	Frequency, duration and intensity of activities (light-moderate-vigorous) in a week
Malkawi et al. [22]	Arabic Preschool Activity Card Sort (PACS)	Jordan	Children with typical development (*n* = 151)	3–6 years of age	Proxy report (by parents)	Interview	Self-care, community mobility, high physical demand leisure, low physical demand leisure, social interaction, domestic, and education	Participation (yes/no). If yes, scale focused on need for assistance/environmental accommodation
Platat et al. [29]	Questionnaire l’Activite Physique en Altitude Chez les Enfants (QAPACE)	United Arab Emirates	Children with typical development (*n* = 79)	6–9 years of age	Proxy report (by teachers)	Paper-and-pencil	Sleep, physical activity at school and home, sedentary activities at home, physical education at school	Total time spent both at school and home
Abd-Elfattah et al. [30]	Perceived Physical Ability Scale for Children (PPASC)	Oman	Children with typical development (*n* = 250)	Average age 10.4 ± 0.63 years	Children	Paper-and-pencil	Self-perception of physical ability	Perceived level of strength, speed, and coordinative abilities
Rostom et al. [25]	Childhood Health Assessment Questionnaire (CHAQ)	Morocco	Children with juvenile idiopathic arthritis (*n* = 60)	4–16 years of age	Proxy report (by parents)	Interview	Activities of daily living	Functional ability (difficulty scale)
Alnahdi et al. [26]	ABILHAND-Kids	Saudi Arabia	Children with cerebral palsy (*n* = 154)	4–15 years of age	Proxy report (by parents)	Paper-and-pencil	Manual ability	Functional ability (difficulty scale)

**Table 2:** Rating of methodological quality of the studies investigating measurement properties of PROMs.

		Reliability			Validity				
Study	Instrument	Internal Consistency	Reliability	Measurement Error	Structural Validity	Construct Validity	Cross-Cultural Validity	Criterion Validity	Responsiveness
Almasri et al. [21]	CAPE-PAC	Very good	Doubtful	Doubtful	Inadequate	Very good	Inadequate	Not investigated	Not investigated
Al-khudair et al. [23]	PEDI	Very good	Doubtful	Doubtful	Inadequate	Very good	Inadequate	Not investigated	Inadequate
Alghamdi et al. [24]	Selfcare-CEDL	Very good	Adequate	Adequate	Inadequate	Not investigated	Inadequate	Not investigated	Not investigated
Regaieg et al. [27]	IPAQ-A	Inadequate	Inadequate	Inadequate	Inadequate	Very good	Inadequate	Very good	Not investigated
Al-Hazzaa et al. [28]	ATLS	Inadequate	Not investigated	Not investigated	Inadequate	Not investigated	Not investigated	Very good	Not investigated
Malkawi et al. [22]	PACS	Very good	Adequate	Not investigated	Inadequate	Very good	Not investigated	Very good	Not investigated
Platat et al. [29]	QAPACE	Inadequate	Doubtful	Doubtful	Inadequate	Not investigated	Inadequate	Very good	Not investigated
Abd-Elfattah et al. [30]	PPASC	Not investigated	Not investigated	Not investigated	Very good	Not investigated	Not investigated	Not investigated	Not investigated
Rostom et al. [25]	CHAQ	Very good	Adequate	Not investigated	Inadequate	Very good	Inadequate	Not investigated	Not investigated
Alnahdi et al. [26]	ABILHAND-Kids	Inadequate	Adequate	Adequate	Very good	Doubtful	Very good	Not investigated	Not investigated

**Table 3:** Rating of psychometric properties of PROMs included in the review.

		Reliability			Validity				Responsiveness
Study	Instrument	Internal Consistency	Reliability	Measurement Error	Structural Validity	Construct Validity	Cross-Cultural Validity	Criterion Validity	
Almasri et al. [21]	CAPE-PAC	+ CAPE: Cronbach alpha 0.61 to 0.83; PAC: Cronbach alpha 0.59 to 0.85	? Not provided	? Not provided	− Principal component analysis; four-item loading (<0.60)	+ MANOVA to determine subgroup differences based on age, gender, disability	? No group analysis for the purpose of cross-cultural validity	? No correlation with gold standard	? Not provided
Al-khudair et al. [23]	PEDI	+ Cronbach alpha 0.87 to 0.98	? Not provided	? Not provided	? Not provided	? Subgroup analysis was carried out but not support construct validity	+ Comparison between US and Saudi children	? No correlation with gold standard	? Not provided
Alghamdi et al. [24]	Selfcare-CEDL	+ Cronbach alpha 0.97 to 0.91	+ ICC 0.99, 95%CI	? SDC 0.29 but MIC not defined	? Not provided	? Not provided	? Not provided	? No correlation with gold standard	? Not provided
Regaieg et al. [27]	IPAQ-A	? Not provided	+ ICC 0.73 to 0.95	? Not provided	? Not provided	? Subgroup analysis was carried out but not support construct validity	? No group analysis for the purpose of cross-cultural validity	? No correlation with gold standard	? Not provided
Al-Hazzaa et al. [28]	ATLS	? Not provided	? Not provided	? Not provided	? Not provided	? Not provided	? No group analysis for the purpose of cross-cultural validity	? No correlation with gold standard	? Not provided
Malkawi et al. [22]	PACS	+ Cronbach alpha 0.859	+ ICC 0.976	? Not provided	? Not provided	+ Subgroup analysis was done to support construct validity	? No group analysis for the purpose of cross-cultural validity	? No correlation with gold standard	? Not provided
Platat et al. [29]	QAPACE	? Not provided	− ICC 0.4 to 0.5	? Not provided	? Not provided	+ Spearman’s correlation coefficients were used to support construct validity	? Not provided	? No correlation with gold standard	? Not provided
Abd-Elfattah et al. [30]	PPASC	+ Cronbach alpha 0.85 to 0.89	? Not provided	? Not provided	+ CFI 0.981 to 0.986	? Not provided	? Not provided	? No correlation with gold standard	? Not provided
Rostom et al. [25]	CHAQ	+ Cronbach alpha 0.90 to 0.98	+ ICC 0.82 to 0.97	? Not provided	? Not provided	+ Spearman’s correlation coefficients were used to support construct validity	? No group analysis for the purpose of cross-cultural validity	? No correlation with gold standard	? Not provided
Alnahdi et al. [26]	ABILHAND-Kids	+ Person separation index 0.93	+ ICC 0.98	? SDC 0.68 but MIC not defined	+ Adequate model fit	+ Spearman’s correlation coefficients were used to support construct validity	+ Comparison between Belgium and Saudi children	? No correlation with gold standard	? Not provided

CFI, comparative fit index; ICC, intraclass correlation coefficient; SDC, smallest detectable change; MIC, minimal important change. “+” sign, sufficient rating; “−” sign, insufficient rating; “?” mark, indeterminate rating.
